# Role of new vaccinators/pharmacists in life-course vaccination

**DOI:** 10.1080/07853890.2024.2411603

**Published:** 2024-10-25

**Authors:** T. Mark Doherty, Lois Privor-Dumm

**Affiliations:** a GSK, Wavre, Belgium; b Johns Hopkins Bloomberg School of Public Health, USA

**Keywords:** Pharmacists, life-course vaccination, vaccinators

## Abstract

**Background:**

Vaccines against diseases such as herpes zoster, pneumococcus and influenza are broadly recommended for older adults, but uptake is frequently low.

**Vaccination Bottleneck:**

Part of the reason may be that access to adult vaccination can be problematic, particularly for minorities and other under-served populations. Potential barriers include complex procedures, limited resources in healthcare systems and lack of structured infrastructure.

**Stress-testing expended vaccination:**

The Covid-19 pandemic necessitated rapid expansion of the infrastructure to deliver adult vaccination, and triggered the use of facilities including pharmacies, schools, faith-based organizations, community organizations, shops and hair salons, drive-through centres and mobile vaccination units.

**Improved adult vaccination system:**

Although many such initiatives were temporary, they demonstrated the principle of effective expansion of adult vaccination and education to a range of new providers and settings. Of these, pharmacist involvement in immunization in particular has consistently been shown to be associated with increased immunization rates.

**Integration of new vaccinators:**

This review discusses results from attempts to expand and simplify the adult vaccination process, potentially allowing vaccination to be initiated by the recipient and completed in a single visit. These studies suggest that expanding adult vaccination access to new providers and/or new settings will require development of an integrated plan for preventive healthcare, covering areas such as setting target coverage rates, financial support, and development of immunization information systems accessible to all vaccination providers to maintain accurate immunization records and support interventions such as reminders.

## KEY MESSAGES

- The expansion of adult vaccination to settings outside of conventional doctors’ offices and hospitals showed that other settings, with appropriate controls, can safely be used for vaccination
- Availability of adult vaccination through a range of providers and settings could simplify the vaccination process, potentially allowing vaccination to be more easily initiated by the recipient
- However, in many countries this potential is not utilized due to the need for changes to basic infrastructure and legislation covering aspects such as vaccine education, payment for vaccination, and access to immunization information systems

## Introduction

The global demographic trend of increasing population age is expected to result in substantial increases in the incidence of many vaccine-preventable age-related infectious diseases. For example, the number of cases of influenza, pertussis, herpes zoster (HZ) and pneumococcal disease in people aged 50 years and over in the United States (US) is projected to increase by 36%, 32%, 31% and 64% respectively, over a 30-year period [1]. Increasing adult vaccine coverage has potential to reduce this disease burden and reduce pressure on limited healthcare resources that can improve population well-being in the future [1], particularly in under-served communities and vulnerable populations. However, vaccines are under-utilized in ageing adults in most countries [2], and the implementation of adult immunization programmes lags well behind that of paediatric programmes [3]. Many vaccines for older adults are given in the context of adult vaccination programmes, including healthcare providers, pregnant women and people with specific underlying conditions. In a survey of World Health Organization (WHO) member states, 38% had no adult vaccination programme in place in 2018 [4], and in a survey of 34 countries many do not provide public financing for at least some basic recommended vaccines for older adults [5]. Even in those countries that do have adult immunization programmes, considerable gaps exist between adult immunization goals and coverage rates achieved, and immunization coverage rates in adults are generally lower (often much lower) than for the same diseases in children [6]. In countries where ageing adult vaccination programmes have been established, uptake rates have been persistently low, with only a few exceptions; in 2018/2019 although all European countries had influenza vaccination programmes, only one achieved the WHO recommended coverage of 75% and the lowest coverage was <1% [2]. Implementation of vaccine programmes depends on many factors, including vaccine financing, government advocacy of adult vaccination, access to vaccines, the existence of a vaccine registry and a reminder system, active recommendation by healthcare professionals, and the level of outreach targeting hard-to-reach populations such as migrants and people with poor literacy [2]. Adult vaccination programmes typically lack the structured approach developed to support paediatric vaccination [7]. Although there are variations between different countries, most paediatric vaccination programmes follow broadly similar patterns with detailed and specific age-based recommendations [8], delivered within a well-established infrastructure for paediatic health such as scheduled well-baby visits. In contrast, adult vaccination is less organized, with wide variations in the vaccinations covered, and few adults have access to routine scheduled healthcare visits that could include vaccination. Infrastructure to address issues of adult vaccination access will also need to include strengthening National Immunization Technical Advisory Groups (NITAGs), advocacy for policy change, intersectoral collaborations and tracking systems to support successful implementation [9].

Global vaccination rates in adults are not consistently reported except for severe acute respiratory syndrome coronavirus 2 (SARS-Cov-2; Covid-19). WHO, the European Centre for Disease Prevention and Control (ECDC), the US Centers for Disease Control and Prevention (CDC) and some high-income countries report on seasonal influenza coverage and sometimes other vaccines including pneumococcal and HZ vaccines, but systems set up for childhood immunization have not translated to the adult vaccine space.

Furthermore, as adult healthcare interactions are commonly concerned with treating symptomatic illnesses, they are not well suited to delivering preventive care such as vaccination. Ageing populations tend to have a higher prevalence of chronic medical conditions requiring medical care, which increases pressure on healthcare resources, particularly in primary care. Existing constraints on time may result in fewer opportunities to integrate vaccination into primary healthcare visits and physicians’ offices often do not stock adult vaccines. In some countries, adults seeking vaccination may need to navigate complex procedures, requiring multiple visits that impose practical barriers to vaccine uptake [10,11]. In the US, adults with health insurance and who had a usual place for healthcare were more likely to be vaccinated than those without [12]. These barriers are further evident among racial and ethnic minorities and other under-served populations [12–14]. But even when infrastructure and resources are present, adult vaccination can also be influenced by population factors, including social attitudes to vaccination and perceptions of the authority of healthcare professionals [15], together with lived experiences [14,16].

The Covid-19 pandemic highlighted the risks of infectious disease in ageing populations, with much higher estimated infection-fatality rates in older adults aged 65–74 years or ≥75 years compared with younger adults [17]. The Covid-19 pandemic illustrated the urgency of developing adult immunization and delivery systems [5], and highlighted the need to strengthen immunization systems for adults [4]. It also illustrated that high levels of vaccine coverage can be attained in older cohorts, given the necessary resources [18].

The objective of this review is to explore barriers restricting access to vaccination in adults, and especially older adults, and to consider whether some of the experience gained from the response to Covid-19 can be applied to improve adult vaccination uptake.

## Adult vaccination ‘bottlenecks’

Vaccination programmes have historically focused on infants and children, and vaccine coverage in adults and older adults is typically lower than coverage in children [19–21]. This reflects the situation that adult vaccination has traditionally not had the same public health priority as paediatric vaccination, and adult vaccination infrastructure has not been as extensively developed [20].

### Primary healthcare providers

Adult vaccination is typically accessed through primary healthcare providers, and the process may require visits to more than one healthcare provider. For example, in a survey of HZ vaccination, which is recommended for older adults and reimbursed under Medicare Part D, in the United States of America (US), 49% of physicians stocked and administered the vaccine in their offices, while 33% of physicians referred the patient to a pharmacy to obtain and administer the vaccine, and 9% referred the patient to another clinic or office [22]. The need for multiple visits and/or a waiting period may act as a barrier to vaccination. Furthermore, in healthcare systems where resources are under pressure, delays and difficulties in obtaining primary healthcare appointments may make it problematic for adults to access vaccination through this channel. Larger practices and health systems may have an easier time managing adult vaccination programmes. One study in Los Angeles County, California demonstrated that larger practices with additional resources to order vaccines and interact with the community, address concerns about hesitancy and cost, and have the space to store vaccines were more likely to vaccinate than smaller practices that did not [23]. In a survey of US physicians, 12% of physicians had stopped administering HZ vaccine in their offices due to cost and reimbursement issues [22]. Reimbursement issues were reported as a barrier to providing office-based HZ vaccination by 52% of respondents, and logistical difficulties such as the need for freezer storage by 16% [22]. A later survey using similar methods found that both were still considered major barriers seven years later by 40% of providers specializing in geriatric care in the US, indicating the persistence of these barriers [24]. In 2023, cost sharing and deductibles were eliminated for adult vaccines covered under Medicare Part D in the US, and vaccine coverage for shingles and tetanus-diphtheria-pertussis (Tdap) vaccines increased substantially in 2023 compared with 2021 [25]. This indicates that removing cost barriers to vaccination can significantly increase coverage in the US, although caution should be employed when extrapolating to other healthcare systems.

### Pharmacists and other providers

Some healthcare systems have recognized the problem of limited resources and started to expand access to at least some adult vaccines through providers outside primary healthcare, most commonly pharmacists, although progress is uneven. As pharmacists have generally been at the forefront of efforts to expand vaccination access, more research has been published for this group than for other providers.

In 2020, approximately one-third of countries surveyed permitted pharmacy-based vaccination, most commonly for adults [26]. Since the pandemic, health departments have been providing vaccines at community-based locations including churches, schools, barber shops and salons, senior housing buildings, and community centres [27–31]. The Baltimore City Health Department offered influenza vaccination clinics in senior housing buildings, and found that residents in buildings with the initiative were more likely to receive the influenza vaccination than those in buildings without the initiative [27]. During the Covid-19 pandemic, research also highlighted the disparities in vaccination, highlighting several examples where vaccination in communities, supported by peer ambassadors or other trusted sources could make a difference [27,28,32].

Many factors may affect the slow and uneven expansion of vaccination to additional providers. Fear of adverse reactions is a commonly reported barrier to adult vaccination [7]; however, this appears to be largely independent of vaccinator/setting/access, which form the focus of this review. Pharmacists and other health professionals (physicians, physician assistants and nurse practitioners) in the US reported barriers to adult vaccination including inadequate reimbursement, lack of staff and lack of vaccine storage and handling facilities, inadequate staff expertise, as well as considering vaccination a low priority, or out of scope for the practice [33]. Health departments have indicated a lack of capacity for partnerships at a community level or lack of staff and resources to meet community needs such as language [34].

A shortage of staff time has been reported as a ­barrier to vaccine administration by pharmacists, particularly in solo practice settings where providing immunization would require temporarily stepping away from dispensary responsibilities [35]. A lack of training in vaccine administration may be a further barrier to vaccine provision by pharmacists, at least as perceived by other healthcare professionals. In a survey in two provinces in Canada, where pharmacists are authorized to give vaccinations, 86% of pharmacists believed they had enough training to give vaccines, while significantly fewer nurses (47%) and physicians (32%) shared this belief [35]. A global survey published by the International Pharmaceutical Federation reported that post-graduate training was available for pharmacists with vaccination roles in 35 countries where pharmacy-based vaccination was permitted [26]. Resistance from other healthcare professionals to accepting the role of pharmacists as vaccination providers may derive partly from a lack of awareness of the immunization training received by pharmacists, although it may also reflect established hierarchies within healthcare systems [35]. Limited acceptance of pharmacists’ role as vaccinators by other healthcare professionals was the joint-most frequently reported barrier to expanding the role of pharmacists in countries/territories where pharmacy-based vaccination is available, along with limited acceptance by governments (61% for both) [26]. The same was true in countries without pharmacy-based vaccination, where limited acceptance by other healthcare professionals was perceived as a barrier in 74%, and limited acceptance by government in 77% [26]. Availability of pharmacy-based vaccination varies widely between countries, as shown by the map in the report published by the International Pharmaceutical Federation in 2020 [26], and may also vary between regions or states within a country.

Difficulties with access to record-keeping systems have also been suggested as a barrier to adult vaccination in pharmacies. In a global survey, almost half (*n* = 16, 47%) of 33 countries and territories with pharmacy-based vaccination did not authorize pharmacists to record vaccination details in a shared vaccination record [26]. In a survey in Canada, a majority of pharmacists had no method of identifying unvaccinated adult patients, and could not always upload to or access patients’ vaccination records in province-wide information systems [35]. In Maryland, the Vaccine Equity Task Force, a collaboration convened by the Governor and run by the Army National Guard, and state and local public agencies and private organizations worked with trusted non-profits, faith-based and community organizations to bring vaccinations closer to the community using non-traditional locations for vaccination clinics including churches, schools, community centers or outdoor locations such as parks or set up near transportation. The task force assisted in the approval process so organizations could order vaccines, plan and conduct clinics and report vaccination into Maryland’s ImmuNet vaccine information system [36]. A similar approach working with trained peer ambassadors to engage with the community, build trust and improve vaccine access was undertaken in under-served communities in Baltimore City [32].

Countries where pharmacists are authorized to immunize have reported improvements in overall coverage and high levels of satisfaction among people vaccinated by pharmacists [37, 38], suggesting that these issues can be addressed. The Covid-19 pandemic, suddenly requiring widespread vaccination of adult populations in a short time frame, necessitated dramatic expansion of adult vaccination access in in many countries.

## The Covid-19 pandemic: stress-testing expanded vaccination access

The vaccine most commonly authorized for administration in pharmacies is influenza, reported by 29 of 31 respondents (94%) in a global survey [26]. This reflects the capacity required to manage annual seasonal influenza immunization programmes for certain population groups. The Covid-19 pandemic required a sudden and dramatic increase in accessibility of adult vaccination, as healthcare systems needed to implement vaccination programmes covering entire adult populations. This resulted in widespread expansion of the settings and practitioners who could provide not only Covid-19 vaccination, but education. Pharmacists were granted wider authority in many countries, including Covid-19 testing and vaccine administration [39], and in Canada seven provinces authorize pharmacy technicians with immunization training to administer vaccines [40].

The expansion of vaccination access in response to Covid-19 included many other settings as well as pharmacies. Drive-through vaccination centres in locations such as sports stadia enabled large numbers of individuals to receive Covid-19 vaccination without having to leave their vehicles [41]. Mobile vaccination teams, which have long been a mainstay of immunization programmes outside high-income countries, improved access to Covid-19 vaccination for under-served populations in the US [42,43]. In the Netherlands, mobile vaccination teams with the capability to administer vaccines on location without the need for an appointment were deployed in buses or pop-up locations to areas with low Covid-19 vaccine uptake [44]. Daily vaccination rates substantially increased during deployment of the mobile units, particularly in rural areas [44]. Mobile vaccination units increased the uptake of a first dose of Covid-19 vaccine by 25% in a study in the UK [45]. A programme in the US partnered pharmacy providers with long-term care facilities to deliver on-site Covid-19 vaccination to residents and staff [46]. New York State in the US temporarily permitted trainee healthcare professionals to administer Covid-19 vaccinations, as a way of increasing the pool of providers and supporting the healthcare system [47], and school nurses administered vaccinations at school-located vaccine clinics across the US [48]. A health insurance plan used data from its provider network to identify potential Covid-19 vaccinators, including nurse practitioners, physicians and physician assistants, together with community pharmacies with active licenses for immunization [49], and a physician assistant developed a programme of on-site Covid-19 vaccination at a clinic for patients with human immunodeficiency virus in California [50].

In addition to traditional health providers offering vaccination, community-based organizations played an important role during the pandemic. Not only did they partner with vaccinators to provide community-based vaccination, but they provided other important services that can help address some of the existing disparities stemming from a lack of access, convenience or trust in the healthcare system [51,52]. Strategies that involve community engagement and co-creation of messaging are more likely to result in greater uptake. Use of microtargeting of specific communities has shown success. In California, Kaiser Permanente identified specific zip codes to target communities with greatest disparities in immunization and was able to achieve a vaccination rate of 81% [53]. In Baltimore City, targeted efforts at a community statistical area level led to increased vaccination rates, with over 80% of individuals over 60 years of age vaccinated, and reduced disparities to just over a 10 percentage point difference in Covid-19 vaccination coverage between black and white individuals [32,54].

The Covid-19 pandemic also highlighted the importance of vaccination education and information campaigns both for the general public and for healthcare professionals, already recognized as important factors in increasing uptake of various vaccines in older adults [55]. In a systematic review of studies worldwide, trust in the Covid-19 vaccine was strongly associated with vaccine acceptance [56], and the two variables most strongly associated with speed of vaccine rollout were education and economic conditions [57]. An analysis of data from 113 countries found that trust in government predicted Covid-19 vaccine hesitancy, and analysis of detailed survey data from seven countries found a robust association between willingness to accept vaccination and trust in health institutions, with the most trusting being 12–20 times more likely to accept vaccination than the least trusting [58]. Health literacy and ability to detect misinformation were positively associated with vaccine acceptance in multiple studies [59,60].

The response to the Covid-19 pandemic, therefore, demonstrated the principle that expansion of adult vaccination to a range of new providers and settings could be effectively achieved. Many of the initiatives were temporary responses to the extraordinary circumstances and would not be practical or cost-effective long-term solutions. Nevertheless, the mass vaccination strategies implemented during the pandemic may provide valuable experience that can be applied to improve adult vaccination uptake in the future.

## Incorporating expanded vaccination access into an improved adult vaccination system

Vaccination in an expanded range of settings, mainly pharmacies, has been available in some countries for several years, and there is evidence that it can improve vaccine access and uptake without compromising patient safety. Seasonal influenza vaccination in the US is available in a range of non-medical settings including supermarkets, workplaces, senior/recreation/community centres and schools [61]. More recently, churches, barber shops and hair salons have been used in the United States as a place for both access to vaccination and education [16,62,63]. A survey of Black barber shops across the US in 2020 demonstrated the value of shops and salons and the role they play in the US Black community through outreach and education. Other initiatives have demonstrated the important role that churches and other community organizations play as trusted messengers during the pandemic, guiding further efforts to expand outreach for Covid-19 in new places [28,31,64]. In a survey of vaccinations administered by immunizing pharmacists at community pharmacies in Western Australia in 2015, a high proportion were in rural and remote areas, suggesting that pharmacy provision facilitated access in these areas, and no major adverse events were recorded [65]. In the US, a large proportion of influenza and pneumococcal vaccines were administered in pharmacy settings between 2018 and 2022 [66]. An analysis of vaccinations carried out at the Walgreens pharmacy chain between August 2011 and July 2012 found that over 30% were administered during non-standard hours, including evenings, weekends and public holidays [67]. In the UK, 39% of annual influenza vaccinations in adults aged 50–64 years not in a risk group were administered in pharmacies in 2021–2022 [68]. In Ireland, the total number of people receiving influenza vaccination increased after the introduction of pharmacy-based influenza vaccination, and in 2014–2015, 23% of people vaccinated at retail pharmacies had not received an influenza vaccine before, indicating that pharmacy vaccination was reaching new sections of the population [69]. This is consistent with a survey conducted at community pharmacy locations in Canada in 2013, in which 25% of respondents vaccinated by pharmacists reported that they were not regular vaccine recipients, and 28% said they would not have been vaccinated this year if pharmacy-based vaccination had not been available [38]. A systematic review of data from randomized controlled trials and observational studies in a range of countries worldwide found that pharmacist involvement in immunization increased immunization rates [70]. In Nova Scotia, Canada, where pharmacists began administering seasonal influenza vaccinations in 2013–2014, a longitudinal study found that immunization rates in adults aged 65 years and over increased from 61.8% in 2012–2013 (the year before pharmacist vaccination was introduced) to 71.6% in 2013–2014 and 73.3% in 2014–2015, suggesting that the expansion of vaccination to pharmacists increased overall uptake [37]. Analysis of data from across Canada indicated that provinces with a policy of pharmacist administration of influenza vaccination had modest increases in vaccine coverage and uptake [71]. The experience with Covid-19 vaccination during the pandemic indicated that increased coverage could be associated with vaccination in other healthcare settings, such as mobile vaccination units [44,45].

Expansion of vaccination to new providers and/or new settings will require countries to integrate expanded vaccination access into an overall national plan for preventive healthcare. A plan may include performance indicators covering areas such as a national vaccination strategy, setting target vaccine coverage rates in specific age groups and monitoring coverage rates achieved [72]. Across 16 countries in Europe, 11 had standardized assessment frameworks for vaccine recommendations, with similar recommendations for universal paediatric vaccinations but more variation in adult vaccines (except for seasonal influenza vaccination in older adults, which was recommended in all countries in the study) [72]. Most (14/16) aimed to report annual vaccine coverage rates, with ten using a centralized registry or electronic vaccination records for childhood vaccinations and seven for other age groups [72]. Vaccine coverage rates tended to decline with increasing age of the target age group; rates were consistently high for infant diphtheria-tetanus-pertussis vaccination (89.1%–98.2%), but more variable for adolescent human papillomavirus vaccination (14.1%–85.9% in 13 countries with data available) and even lower for older adult seasonal influenza vaccination (4.3%–71.6% in 14 countries with data available) [72]. A study of factors affecting seasonal influenza vaccination coverage rates in elderly populations in Europe found that the presence of national systems monitoring coverage and sending personal letters inviting people to receive free influenza vaccine were associated with higher coverage rates [73]. Coverage rates for influenza vaccination are below target in many countries around the world [74]. In a global survey of 194 WHO member states, the 59% which reported having a national influenza immunization policy tended to be wealthier, to have introduced more new vaccines, and to have NITAGs [75].

Financial incentives for vaccine recipients and healthcare providers may also help to improve vaccine coverage rates [55]. Some countries offered shopping vouchers and discounts or cash payments to encourage individuals to receive the Covid-19 vaccine [76]. In a survey of vaccination policies in 16 European countries, providing incentives for healthcare workers to immunize patients was associated with numerically higher coverage rates for seasonal influenza vaccination in older adults, although the effect was modest and did not reach statistical significance [73]. Similar results were found in an updated survey a few years later [77]. Incentives can take several forms, which may have different effects on behaviour. For example, fee for service provides an incentive to increase activity for reimbursed services (e.g. by increasing the volume of immunization), but may act as a disincentive to other activities such as patient education [78]. Payment for performance for family doctors has been shown to increase childhood immunization coverage in Estonia [79] but may be perceived as limiting professional autonomy [78]. It is important to consider the impact of expanded vaccination on the earnings of healthcare providers, as cost and reimbursement issues have been noted as barriers to provision of vaccinations [22,33]. Developing objectives and incentives that apply fairly across the healthcare system is a necessary first step to ensure fair reimbursement and to prevent the development of perverse incentives.

Effective immunization information systems (IIS) for adults that track vaccination coverage will also be important to support expanded vaccination programmes. Accurate centralized information is essential for an effective reminder system, particularly for adults who may see a range of healthcare providers and receive vaccination in several settings [80]. Individuals may not always have accurate information on their vaccine status. In a study in Lyon, France, a substantial proportion of people attending private laboratories for tests of any kind mistakenly thought that they were up-to-date with vaccinations for pertussis, diphtheria, tetanus and poliomyelitis [81]. Most countries in the European Union/European Economic Area have an IIS in operation or in development [82]. IIS can support a variety of interventions to improve vaccine coverage, such as reminder and recall for individuals, provider assessment and provider reminders [83]. Reminders, such as telephone calls, letters, postcards or text messages, can increase immunization rates in children, adolescents and adults [84]. In a systematic review of studies in influenza vaccination uptake in people aged 60 years and older, simple reminders (e.g. letters), personalized reminders and health risk appraisal plus vaccine recommendations improved vaccination rates [85]. An integrated IIS accessible to all vaccine providers will be needed to maintain accurate and comprehensive immunization records and support interventions such as reminders. This would also address some potential concerns about accurate vaccination recording by new providers. However, this requires allowing non-traditional vaccinators access to at least some parts of patients’ records. Some countries, such as Denmark, Iceland, Malta, Norway, mainland Portugal and Andalucia (Spain) have systems that can be accessed by healthcare personnel to record vaccine administration in real time throughout the life course [82], while many other countries do not permit access to some vaccine providers such as pharmacists [26,35].

Widening the range of vaccine providers and settings also has the potential to simplify the process of immunization for patients. In a study in the US, 36% of physicians referred the patient to a pharmacy to obtain the vaccine followed by administration in the physician’s office [22], a process requiring three separate visits. Vaccination at a pharmacy or other community based location removes the need for a return visit to the physician’s office, and if the patient can initiate vaccination by going directly to the pharmacy or the place they are to be vaccinated the process can be completed in a single visit. In Ontario, Canada, pharmacists were authorized to administer seasonal influenza vaccinations from the autumn of 2012, and uptake in the first year was more than double the provincial government target [38]. A survey of people vaccinated at four community pharmacies in Toronto, Ontario, in 2013 found that 92% reported being ‘very satisfied’ with the service, and 46% commented on its convenience [38]. In the US, states had significantly higher adult seasonal influenza vaccination rates after changes in policy to permit pharmacists to administer seasonal influenza vaccination, with the effect increasing over time [86]. Other settings may also offer potential for improving adult vaccination uptake. An on-site clinic in a homeless shelter in the US offering HZ vaccination on Shingles Immunization Days achieved an immunization rate of 38% [87] which is especially encouraging in a cohort that is considered hard to reach *via* traditional healthcare services. In a more traditional setting, a study of catch-up vaccination of adults aged 65 years and over admitted to hospital in central France found that diphtheria-tetanus-inactivated polio virus vaccine coverage increased from 56% to 81% [88].

**Figure 1.**
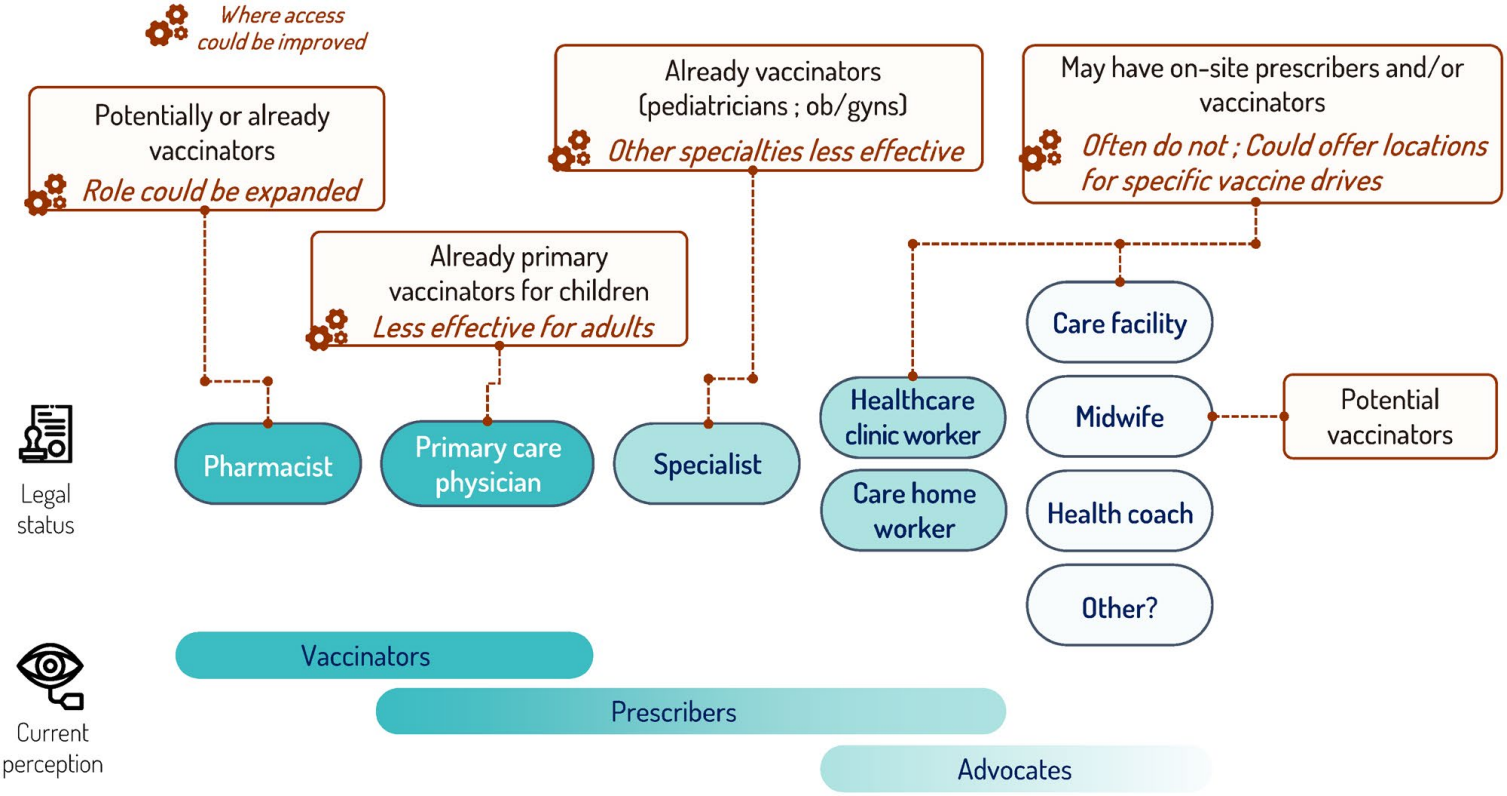
Potential roles for new vaccinators.

The Covid-19 pandemic experience showed that vaccination can be expanded to other settings beyond pharmacies, such as long-term care facilities [46] and mobile vaccination units [45]. Mobile vaccination units may be especially valuable in medically under-served populations where vaccine uptake is generally lower than average [42–44]. Vaccination providers and prescribers in a range of settings need to be integrated into a national vaccination plan to maximize the potential benefits. Figure 1 illustrates some of the vaccine providers and settings that could be developed to improve access to adult vaccination. While in some regions of Canada midwives are already involved in vaccination [89], this direct involvement mostly remains limited.

## Models for integration of new vaccinators into adult vaccination programmes

Two models could be considered to integrate new vaccinators into adult vaccination programmes. In the first, prescribers act as gatekeepers to vaccination. These are exclusively medically trained staff, mostly physicians. Prescribers may also be vaccinators, and vaccinators may also include a wider range of appropriately trained and equipped healthcare professionals in a variety of settings such as pharmacies, long-term care facilities and community-based settings including community centres, churches, barber shops and salons or mobile units where trusted messengers coordinate with vaccinators to bring services closer to them. This can also be combined with education, as many health providers have an acute care rather than prevention focus. This model would be the easiest to integrate into most existing systems, since it mimics that already in use for most medicines: prescribing is done by doctors, but a prescription can be filled in the doctor’s office, or in other settings.

In the second model, a prescription is not necessarily a requirement for vaccination. Unlike other medications, many vaccines are effectively approved and prescribed for an entire eligible patient population and are available to all individuals in that cohort unless there is a specific contraindication. Many universal infant vaccination programmes operate on this principle. Some countries already operate seasonal influenza vaccination using this approach, allowing vaccinating pharmacies to vaccinate anyone who is eligible and wants to be vaccinated without a prescription. This model is the simplest for the patient and its convenience may mean it is the most effective at expanding coverage. However, to be viable it requires access by vaccinators to patient records to ensure that eligibility and potential contraindications can be checked, and that necessary guidelines around reimbursement, responsibility and liability are in place.

## Conclusions

Adult vaccination coverage rates are generally lower than for the same diseases in children. Barriers to adult vaccination vary between countries and healthcare systems, and may include limited healthcare resources, the need for multiple visits and reimbursement issues. While it would be interesting to compare barriers to vaccination across different countries, the lack of published comparable data and significant differences in funding and access currently makes meaningful comparisons difficult. This is an area that could usefully be investigated in future research. Additionally, pharmacists have been at the forefront of efforts for expanded vaccination access prior to and following the COVID-19 pandemic.

The response to the Covid-19 pandemic required rapid expansion of adult vaccination, and proved that expansion of vaccination access to many new providers and settings, including those with a primary focus both inside and outside healthcare, can be done successfully. Both adult vaccination and education could feasibly be extended to a range of new providers and settings on a more permanent basis. Expanded vaccination access will need to be integrated into an overall national plan for preventive healthcare. This could be done using a model in which prescribers act as gatekeepers for vaccination and the vaccination can be provided in a range of settings, or an alternative model allowing vaccination to be available for anyone who is eligible and wants to be vaccinated without the need for a prior prescription.

## Data Availability

Data sharing is not applicable to this article, as no new data were created or analyzed in this study.

## Abbreviations

HZ: herpes zoster
IIS: immunization information systems
SARS-Cov-2: severe acute respiratory syndrome coronavirus 2
UK: United Kingdom
US: United States of America
WHO: World Health Organization
